# Expression alteration of microRNAs in Nucleus Accumbens is associated with chronic stress and antidepressant treatment in rats

**DOI:** 10.1186/s12911-019-0964-z

**Published:** 2019-12-19

**Authors:** Weichen Song, Yifeng Shen, Yanhua Zhang, Sufang Peng, Ran Zhang, Ailing Ning, Huafang Li, Xia Li, Guan Ning Lin, Shunying Yu

**Affiliations:** 1 Shanghai Key Laboratory of Psychotic Disorders, Shanghai Mental Health Center, Shanghai Jiao Tong University School of Medicine, Shanghai, China; 2 School of Biomedical Engineering, Shanghai Jiao Tong University, Shanghai, China

**Keywords:** Stress, Antidepressant, microRNA, Small RNA-sequencing, Nucleus Accumbens, Escitalopram, Anhedonia, Ras-signaling pathway

## Abstract

**Background:**

Nucleus Accumbens (NAc) is a vital brain region for the process of reward and stress, whereas microRNA plays a crucial role in depression pathology. However, the abnormality of NAc miRNA expression during the stress-induced depression and antidepressant treatment, as well as its biological significance, are still unknown.

**Methods:**

We performed the small RNA-sequencing in NAc of rats from three groups: control, chronic unpredictable mild stress (CUMS), and CUMS with an antidepressant, Escitalopram. We applied an integrative pipeline for analyzing the miRNA expression alternation in different model groups, including differential expression analysis, co-expression analysis, as well as a subsequent pathway/network analysis to discover both miRNA alteration pattern and its biological significance.

**Result:**

A total of 423 miRNAs were included in analysis.18/8 differential expressing (DE) miRNA (adjusted *p* < 0.05, |log2FC| > 1) were observed in controls Vs. depression/depression Vs. treatment, 2 of which are overlapping. 78% (14/18) of these miRNAs showed opposite trends of alteration in stress and treatment. Two micro RNA, miR-10b-5p and miR-214-3p, appeared to be hubs in the regulation networks and also among the top findings in both differential analyses. Using co-expression analysis, we found a functional module that strongly correlated with stress (*R* = 0.96, *P* = 0.003), and another functional module with a moderate correlation with anhedonia (*R* = 0.89, *P* = 0.02). We also found that predicted targets of these miRNAs were significantly enriched in the Ras signaling pathway, which is associated with both depression, anhedonia, and antidepressant treatment.

**Conclusion:**

Escitalopram treatment can significantly reverse NAc miRNA abnormality induced by chronic stress. However, the novel miRNA alteration that is absent in stress pathology also emerges, which means that antidepressant treatment is unlikely to bring miRNA expression back to the same level as the controls. Also, the Ras-signaling pathway may be involved in explaining the depression disease etiology, the clinical symptom, and treatment response of stress-induced depression.

## Background

Major Depressive Disorder (MDD) is a chronic, severe disorder lacking reliable biomarker and mostly relying on the symptom-based diagnosis [1]. Due to the limited understanding of disease mechanism, precise diagnosis [2] and personalized treatment [3] of the disorder is still lacking. It has been discovered that chronic stress is associated with a disturbance in cognition, and daily function of MDD patients, and plays a vital role in the pathology of depression [4]. Malfunction of the hypothalamic-pituitary-adrenal axis and sympathetic nervous system induced by stressful life events is believed to be associated with depression [5]. However, the molecular mechanism underlying stress pathology is not fully understood.

The epigenetic mechanism in depression, a popular model of Gene-Environment interaction, has gained much attention in psychiatric researches [6]. microRNA (miRNA), a type of 18–24 nucleotides-long non-coding RNA that negatively regulates the expression of genes, is of particular interested. By formation of the RNA-induced silencing complex (RISC), miRNA influence both translation and levels of message RNA [7]. Role of miRNA in the pathology of depression has been emphasized [8]. Various studies focused on the difference of miRNA expression between depression patients and control, both in the brain and in peripheral tissue [8–11], which revealed a significant difference of patterns of miRNA expression in depression patients. Research also suggests that the miRNA expression pattern may serve as a biomarker for diagnosis of depression [9] or response of antidepressant [10, 11]. To achieve these goals, a comprehensive understanding of the impacts of depression/antidepressant on miRNA expression is required.

Nucleus Accumbens (NAc) plays a vital role in the circuitry of reward and stress [12], and transcriptomic differences of this area contribute to the susceptibility to stress [13] and social defeat [14]. Notably, the sex difference of vulnerability to stress is associated with both mRNA and miRNA expressing patterns of NAc [13, 15]. Epigenetic regulation in NAc may also influence stress-induced depression [16]. Mesocortical Circuit, which has a close relationship with NAc and participates in the hedonic procedure, shows alteration of miRNA expressing during stress [17]. Other studies suggest that miRNA regulates the function of NAc during alcohol [18] or substance abuse [19]. However, scientists seldom focus on miRNA change of NAc during chronic stress; research on response to an antidepressant is also lacking.

**Fig. 1:**
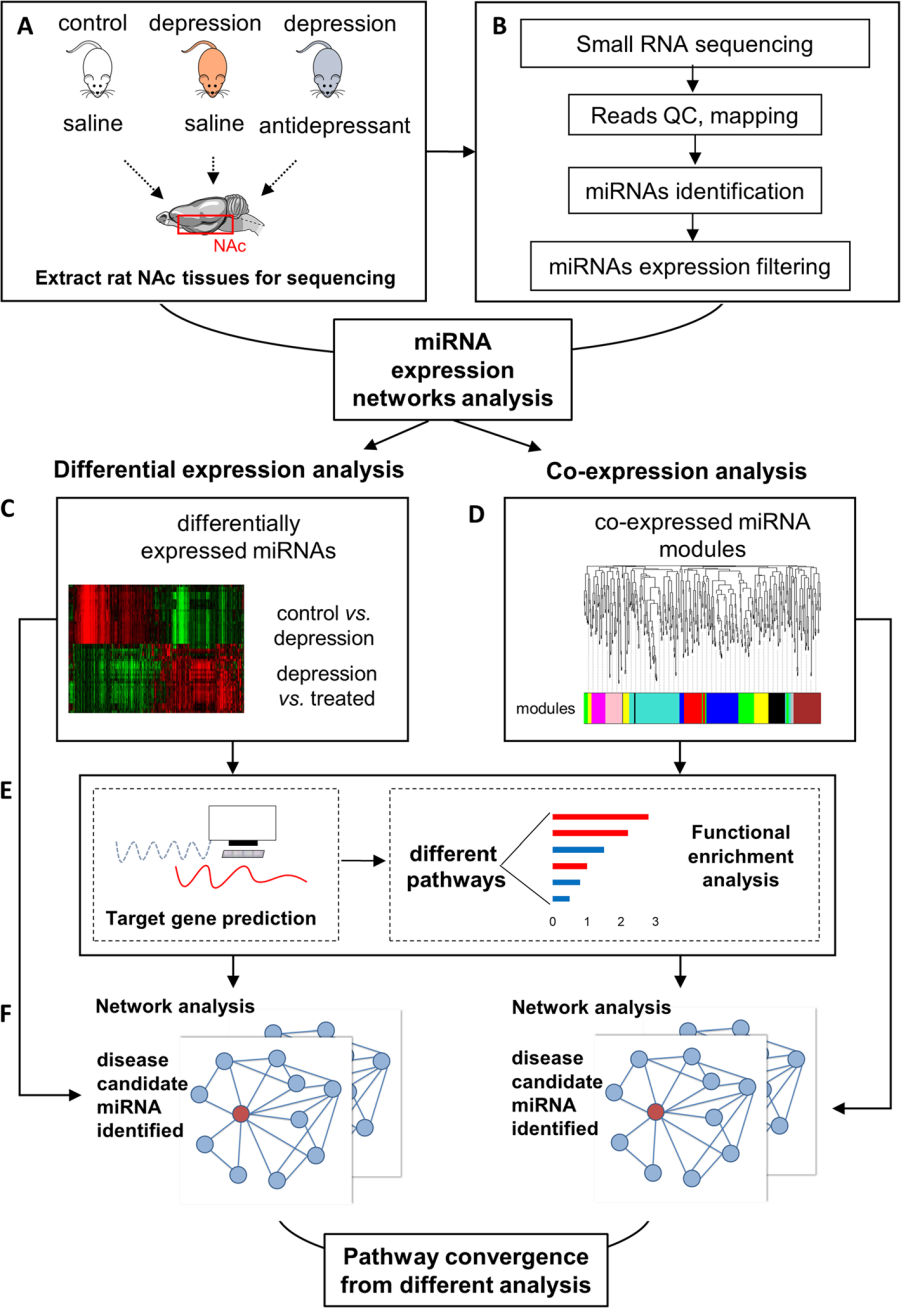
The flow chart of the study

In the current study, we set out to address two questions: 1) how does NAc miRNA expression alter during chronic stress? How does antidepressant reverse this alteration? 2) What are the key pathways and molecules in stress- and antidepressant-associated miRNA networks? We studied NAc from three groups of rats: control, stress, and stress+ antidepressant. To induce depression-like behaviors in mice, we used an established protocol, chronic unpredictable mild stress (CUMS) (Fig. 1a) [20]. We analyzed small RNA sequence data from both of these groups (Fig. 1b). The underlying biological significance is also studied by downstream analysis. The fact that there exist complex many-to-many relationships between miRNA and mRNA makes it difficult to highlight the critical molecules in high-throughput data analysis. To overcome this difficulty, we built up an integrative pipeline which consists of differential expression analysis (Fig. 1c), weighted co-expression analysis (Fig. 1d), and downstream pathway & network analysis (Fig. 1e and f). In differential expression analysis, we focus on critical miRNAs with profound expression alteration; in weighted co-expression analysis, genes being regulated by a group of miRNAs with subtle yet convergent alteration are highlighted.

## Results

### Identification of brain NAc tissue expressed miRNAs from different rat model groups

In our previous work [20], we constructed two successful animal models to simulate the human depression clinical manifestation in two different scenarios: 1) “case” group - rats with depression symptoms by stress; 2) “treated” group - rats with depression symptoms underwent antidepressant (Escitalopram) treatment (Fig. 1a). Using these models, we performed the behavior studies and observed that case rats with CUMS had developed weight loss, anhedonia, and anxiety-like behaviors. Escitalopram can reverse the symptoms of anhedonia but had little influence on weight loss and anxiety-like behaviors (Additional file 1 a–b).

To quantify the miRNAs expression in Nucleus Accumbens (NAc) during stress and Escitalopram treatment, we performed the small RNA sequencing on the NAc tissue from brains of all three groups of rats. A total of 48 million raw reads were obtained. After quality control, removal of barcodes and low-quality reads (reads with any ambiguous base, reads length < 18 bases), about 71% of raw reads were retained as high-quality reads and were used in the following steps (Fig. 1b). Approximately 12 million reads were successfully aligned and mapped to a total of 586 mature miRNAs recorded by miRBase [21] (Fig. 1b, Additional file 2). Before analyses, we summed raw counts across all samples and removed the bottom 25% of miRNA avoid the noise of low read-counts. These procedures left us 423 miRNAs for downstream analyses.

### Escitalopram can reverse stress-induced miRNA abnormality

**Fig. 2:**
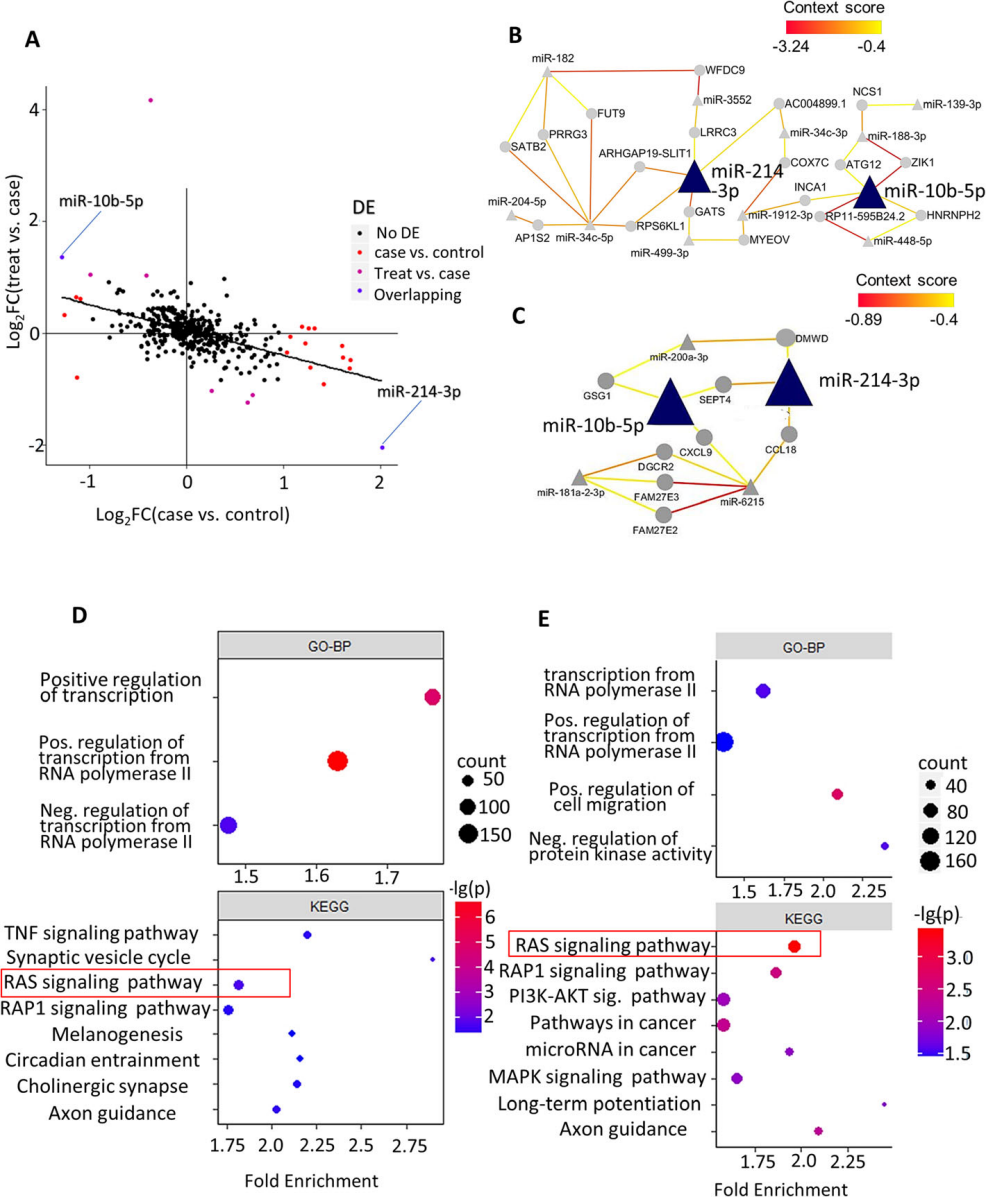
differential expressing analysis and downstream pathway/network analysis. **a** Tendency of miRNA alteration.x-axis:log2(fold change) in case vs. control; y-axis:log2(fold change) in treat vs. case. Colored dots represent miRNAs that reach criteria of significant differential expression (FDR < 0.05, absolute log2FC > 1). **b** top GO-BP and KEGG pathways for case vs. control DE-miRNA targets. **c** top GO-BP and KEGG pathways for treat vs. case DE-miRNA targets. **d** Network analysis for case vs. control DE-miRNA. **d** Network analysis for treat vs. case DE-miRNA. For (**d**, **e**), triangles represent miRNAs and circles represent transcription products of genes. A straight line means that the mRNA is predicted to be regulated by the miRNA, and the color of the line represents the context score of this prediction (see method). Red lines represent a lower score, which means the prediction is highly credible

With the miRNA transcriptome of NAc being quantified, we sought to find miRNAs that significantly altered in case (stress) or treated rats by the differential expression (DE) analysis. Eighteen miRNAs exhibited differential expressions with statistical significance when comparing case to control (13 down-regulated and five up-regulated), while 56% of them (10/18) remained significant when comparing treated to control. Next, we observed that the majority of these 18 DE miRNAs (78%, 14 out of 18) exhibited an opposite DE change when comparing treated to the case (Fig. 2a, Table 1), which indicate the effectiveness of the Escitalopram treatment. Eight DE miRNAs were observed when comparing treated to the case. Not surprisingly, all of them had a reverse alteration when comparing case to control. Furthermore, we observed that two miRNAs, miR-10b-5p and 214-3p, showed DE in both comparisons (case vs. control and case vs. treated), indicating their potential roles in both etiology and treatment of depression.

**Table 1:** Top DE-miRNAs from Case vs. control (18 DE-miRs total) and Treatment vs. case (8 DE-miRs total)

Case vs. control				Treatment vs. case			
miRNA	log2 FC	Relevance to Neuropsychiatric disease	Ref	miRNA	log2FC	Relevance to Neuropsychiatric disease	Ref
miR-1298	1.70	NA		miR-7a-5p	−1.10	NA	
miR-139-5p	1.28	May Marks sensitivity to stress	[22]	miR-181a-2-3p	1.05	Related to Cerebral cavernous malformations	[23]
miR-139-3p	1.41	↓by Nerve Growth Factor treatment	[24]	miR-200a-3p	1.02	Mediate antidepressant-like effects in stressed rat	[25]
miR-34c-5p	1.23	↓in blood&brain of MDD patients	[26, 27]	miR-214-3p	−2.05	adjusts Depressive-like Behaviors	[28]
miR-448-3p	1.61	NA		miR-32-3p	−1.24	NA	
miR-204-5p	1.07	Suppressed by morphine	[29]	miR-6215	4.16	NA	
miR-10b-5p	−1.29	Related to Parkinson disease	[30]	miR-653-5p	− 1.02	NA	
miR-214-3p	2.02	adjusts Depressive-like Behaviors	[31]	miR-10b-5p	1.36	Related to Parkinson disease	[30]

To further characterize the general trend of miRNA expression alteration, we performed a linear regression analysis on the fold change (FC) of DE. We observed that FC of miRNA expression between the case vs. treated was adversely correlated with that of case vs. control (slope = − 0.45, *P*-value = 2.6 × 10^− 28^, Fig. 2a), suggesting that antidepressant can dramatically reverse stress-induced miRNA alteration in NAc, but not entirely.

### The Ras-signaling pathway was influenced by stress and antidepressant treatments

To further explore biological significance beneath expressions abnormality of Nucleus Accumbens miRNAs, we predicted the target genes of these differentially expressed miRNAs by Targetscan [32] and performed KEGG-enrichment and GO pathway analyses on these putative targets (see METHOD). Since rats from treatment group encountered both CUMS and antidepressant treatment, we reasoned that by comparing the treated rat group directly with the control group could introduce risk factors that would be complex and difficult to be identified. Thus, we decided to perform the following analyses focusing on case Vs. control, and treatment vs. case.

Using Database for Annotation, Visualization and Integrated Discovery (DAVID) [33], we revealed that several pathways related with neuronal functions, such as Synaptic vesicle cycle, Ras signaling pathway, and axon guidance, were enriched by the putative targets of DE miRNA (Fig. 2b, c). We observed that the Ras signaling pathway was the most significantly enriched pathway for putative targets of DE miRNA in case vs. treated (Fig. 2c) (Benjamin adjusted *p* = 3.7 × 10^− 4^). This pathway was also significantly enriched by the putative targets of DE-miRNAs in case vs. control comparison (Fig. 2b). Another significant term shared by two comparisons was axon guidance, which plays a critical role in both the development of the nervous system and psychiatric pathology.

### Broad influence of miR-10b-5p&214-3p in depression and antidepressant treatment

We hypothesized that miRNAs miR-10b-5p and miR-214-3p have an important role in regulating the NAc miRNA network in both case and treated rats, given that they were observed to be significantly differentially expressed in both case vs. control and case vs. treated. To interrogate this, we started by constructing two miRNA-gene regulation networks centered on DE miRNAs with their putative targeted genes, one for case vs. control and the other for case vs. treated. We next performed the systematic network analyses on them. Both miRNA networks formed tightly connected graphs (Fig. 2d, e). Two overlapped miRNA, miR-10b-5p and miR-214-3p, appeared to be hub nodes with a max of 5 partners in both networks. In the network of control vs. depression DE-miRNAs, no other miRNA except miR-34c-5p (6) has more neighbors than miR-10b-5p and miR-214-3p. Furthermore, the weighted context score (represented by colors of the line) of these two miRNAs is relatively low, which means that the predicted targets of them are highly confident.

### WRCNA analysis revealed modules associated with stress and anhedonia

**Fig. 3:**
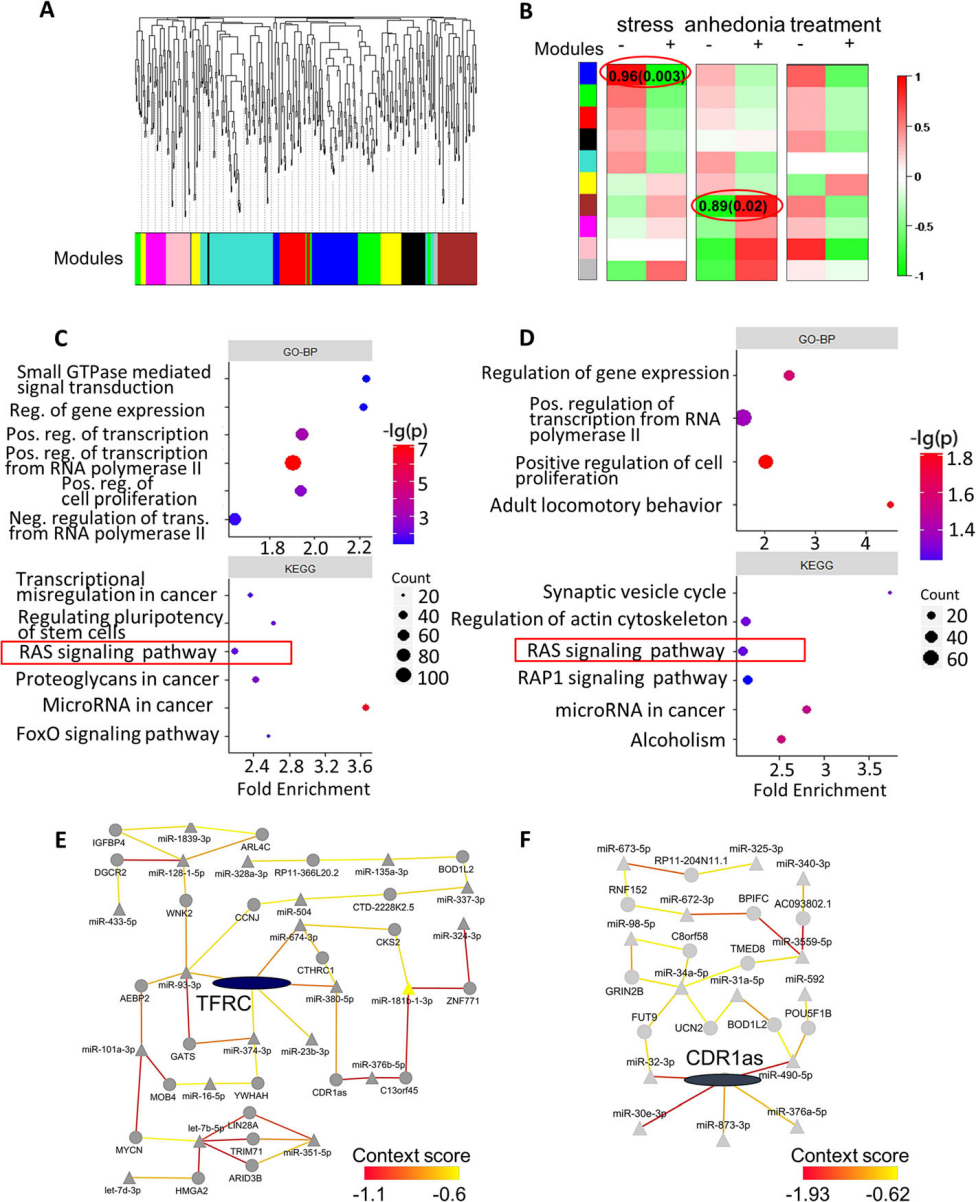
WRCNA analysis and downstream pathway/network analysis. **a** Dendrogram of co-expression analysis. **b** Module-trait (stress, anhedonia, and treatment) correlation. Pearson correlation of module eigengene (see method) and the trait is showed as color in each grid. Two results with *p* < 0.05 are shown as correlation(*p*-value) in corresponding grids and are highlighted by red circles. **c** Pathway analysis for M1. **d** Pathway analysis for M7. Same as Fig. 2b and c. e: Network analysis for M1. **f** Network analysis for M7. Same as Fig. 2d and e

Besides sets of miRNAs being significant expression altered in depression disorder, we also hypothesized there were genes being regulated by a group of miRNAs with subtle but convergent changes and could be linking to specific clinical behavioral abnormality in the system and network level. To explore this concept, we applied the famous Weighted Gene Co-expression Network Analysis WGCNA [34] tools on miRNA data, namely WRCNA, and performed subsequent module-trait correlation analysis (see METHOD). We obtained ten modules by WRCNA (Fig. 3a). Using module-trait correlation analyses, we found that the dark blue module (M1), consisting of 56 miRNAs, showed a strong positive correlation with exposure to chronic stress (Pearson correlation coefficient (PCC) = 0.96, *P* = 0.003, Fig. 3b). Another module, brown module(M7) of 55 miRNAs, also showed a positive correlation with anhedonia (PCC = 0.89, *P* = 0.02). However, we obtained no significant correlation between any module and treatment.

To gained biological insights of M1 and M7, we carried out miRNA-gene regulation network analyses and pathway analysis on them. In network analysis, we focused on miRNAs within these two modules and predicted all the targeted genes using TargetScan [32]. Next, we filtered out all putative miRNA-gene interactions with a context score > − 0.6 (see METHOD). We also removed genes being predicted to be the target of only one miRNA. Isolated networks were also discarded.

### The Ras-signaling pathway was associated with stress and anhedonia

In pathway analysis for putative targets of miRNA in M1(Fig. 3c), we again observed enrichment in the Ras signaling pathway. This was in agreement with results from DE analysis. As for M7 module, we found only nominal significant results, partly due to the limited number of putative target genes (Fig. 3d). Nonetheless, we could observe enrichments in the Ras signaling pathway besides other brain-relating pathways, such as synaptic vesicle cycle and alcoholism. Predicted target genes for both modules also enriched in the term “positive regulation of transcription from RNA polymerase II promoter” and other terms related with regulation of transcription, suggesting an alteration of transcription caused by stress pathology.

### TFRC and CDR1as implicated in stress and anhedonia

To investigate which hub targets in M1 could be associated with stress and M7 with anhedonia, we further analyzed the miRNA-gene regulation network of M1 and M7. We found that a gene, TFRC, connecting with five miRNA regulators in M1 module, comparing to all other target genes in M1 having < 3 regulators. This highlighted the potentially important role of TFRC in chronic stress, regardless of antidepressant treatment. In the regulation network of M7 module, gene CDR1as had the most five putative regulators with among the lowest context score (Fig. 3e, f). Another interesting finding was that miR-34c-5p, one of the top miRNAs with differential expression in case vs. control (Table 1), had more putative targets than all other miRNAs, suggesting the misregulation of these two molecules in NAc may play a role in anhedonia.

## Discussion

In this study, we demonstrated that Escitalopram could dramatically reverse both stress-induced anhedonia (represented by sucrose preference, Additional file 1 b) and dysregulation of miRNA expression caused by chronic stress (Fig. 2a). Similar to our results (Fig. 2a), Bagot et al. [35, 36] also discovered that antidepressant treatment could significantly reverse stress-associated gene expression alteration in multiple brain regions, including NAc. In their study, up-regulating genes during ketamine usage profoundly overlapped down-regulating genes during the depression in NAc, and vice versa. Together with our results on miRNA, one could infer that drug treatment can reverse pathological gene-expression regulation, at least in part, by reversing pathological miRNA regulation. However, we should also notice that Escitalopram cannot reverse this disruptive expression pattern exactly back to normal. Actually, more than half (10 out of 18) of DE-miRNA in case vs. control were still differentially expressing in treatment vs. control comparison.

On the other hand, Escitalopram does not merely act on the abnormality of miRNA caused by depression; furthermore, this treatment creates novel alterations of miRNA expression, which differs from both healthy and depressed states. As we can see, only a quarter of treatment-related DE-miRNAs (2/8) overlaps with depressed-related DE-miRNAs. Consistent with this idea, Zhou M [37] studied the miRNA alteration in rats hippocampus caused by CUMS, whereas Miao N [38] and Yang X [39] studied alteration caused by Fluoxetine/Ketamine in the same region, and their results have little overlap. Pan B [40] carried out a similar study with Duloxetine, and the little converging result is found despite miR-124, a well-studied miRNA involved in depression [41] and antidepressant treatment [42]. These results in part suggest that antidepressant treatment does not totally reverse chronic stress pathology, but rather cause novel alteration. Similar to our study, Miao N [38] found enrichment of target antidepressant-altered miRNA in axon guidance pathways, emphasizing roles of this pathway in antidepressant therapy.

One of our key findings in pathway enrichment analysis is Ras-signaling pathway. Predicted targets of miRNAs that were associated with stress, treatment, and anhedonia showed consistent enrichment in this pathway. For putative targets of the case versus treatment DE-miRNAs, the *p*-value of enrichment was particularly small (Fig. 2c). It could be inferred that no matter antidepressant is present or not, chronic stress can always induce dysregulation in Ras system. Renin-angiotensin system (RAS), a critical molecular axis in stress procedure, play an essential role in the regulation of normal brain function. Serotoninergic [43] and noradrenergic [44] neurons, which are densely linked with effect and stress-associated disorder, have been demonstrated to be regulated by members of RAS system like AngII, AT1 and HPA axis [45]. Genetic studies have found the causal relationship between polymorphism on genes that encode angiotensin-converting enzyme (ACE) and risk of MDD [46], the severity of depressive symptom [28] and outcome of antidepressant treatment [47]. These findings support our suggestion that the Ras system is associated with both stress exposure, antidepressant usage, and anhedonia. What’s more, the antagonist of RAS system can trigger an antidepressant-like effect both in human [48] and rats [49], which suggest that RAS system may be a candidate therapeutic targets in depression treatment.

In our study, miR-10b-5p and miR-214-3p appear to be critical molecules of stress pathology and antidepressant treatment. They show significant DE in both case Vs. control and treat vs. case (Table 1) and play a central role in both network analyses (Fig. 2d–e). Deng et al. [31] demonstrated that miR-214-3p is significantly up-regulated in the prefrontal cortex in Chronic Social Defeat Stress Mice. Other studies confirmed that miR-214-3p could regulate cerebral cortex development [50] and dendritic development [51]. On the other hand, the roles of miR-10b-5p in psychiatric disorder is not yet well studied. Our results suggest that these two microRNA may be a candidate molecule for further research on stress and antidepressant mechanism.

By analyzing relations between co-expression modules and traits (Fig. 3b), we demonstrate that M1 & M7 miRNAs are profoundly association with stress & anhedonia. *TFRC* is one of the hub genes in the miRNA-gene regulation network of M1 (Fig. 3e). *TFRC* encodes Transferrin receptor, which binds to diferric transferrin and serves as a transporter of the extracellular iron-transferrin complex. *TFRC* impacts a wide range of neuronal function and activity, such as long-term potentiation and AMPA receptor distribution [52], motor coordination and trafficking of mGlu1 [53], etc. To our knowledge, no solid evidence has been validated for the role of *TFRC* in psychotic disease. However, 3q29, the chromosome band that harbors the *TFRC* gene, is a hotspot in psychiatric genetic studies [54]^.^ Researchers confirmed association between structural variants in 3q29 and schizophrenia [55],bipolar disorder [56], autism [57] and other neuro-developmental disorders [58]. Since most of these structural variations cover the *TFRC* gene, we can infer that *TFRC* abruption may be involved in the psychotic disease.

One limitation of our study is that we fail to clarify the miRNA mechanism behind antidepressant treatment in NAc. Our DE analysis found only 8 DE-miRNAs in case versus treatment, and WRCNA also failed to find any module associated with treatment. This may result from the relatively short time course of drug treatment, and the effect of Escitalopram may not be fully onset. Another important reason is our small sample size. Further research with more samples is needed to discover the NAc miRNA network associated with Escitalopram.

## Conclusions

We find that Escitalopram usage can profoundly reverse the behavioral as well as NAc miRNA abnormality induced by chronic unpredictable mild stress. However, novel miRNA alteration that is absent in stress pathology also emerges, which means that antidepressant treatment is unlikely to bring miRNA expression exactly back to normal. Renin-angiotensin system takes parts in both onsets of depression, the emergence of anhedonia, and response to an antidepressant. We also conclude that in NAc, miR-214-3p and miR-10b-5p are significantly regulated by both stress exposure and antidepressant usage. These results suggest that we should focus on them for further investigation of depression.

## Methods

### Animal models

A total of 24 adult male Sprague-Dawley rats, aged ranging from 6 to 8 weeks, weighted 200~250 g, were obtained from Department of Experimental Animals, East China Normal University [SYXK (HU)2010–0002], and all animal experiments were supervised by them. Rats were randomly selected to control group (8 rats) and CUMS group (16 rats). CUMS group was further separated into escitalopram-treatment and no-treatment group (8 each). Unexpectedly, one rat in the control group died at week 5, thereafter *n* = 7.

### Chronic unpredictable mild stress (CUMS) and drug treatment

To establish a stress-induced depression model, we applied an improved CUMS procedure as previously described [20]. In brief, after 8-days long placation and adaptation to the environment, eight rats went through 42-days unpredictable stress procedure. The body mass, locomotion behavior, and sucrose preference were measured in day 7 and 8. Body mass was measured every 7 days. Rats in the control group lived without following stresses. For each of the 42 days, two of the following procedures were randomly chosen for CUMS group: wet cage, food exclusion, water exclusion plus Empty bottle stimulation, restraint movement, clip tail, icy water swimming, co-feeding, and stranger. 2 ml of saline Escitalopram solution (10 mg/kg) or saline alone were given to the treatment group or depression/control group via Intraperitoneal injection daily during the time of CUMS experiment.

### Behavioral tests

All rats went through adaptations to open fields and sucrose on the day after unpredictable stress procedure end. Two behavioral tests were taken to see if CUMS group developed depressive-like behavior:

#### Sucrose preference test

In the first day, two bottles of sucrose solution (100 ml, 10%) were provided to all groups. In the second day, one bottle of 100 ml water and one bottle of 100 ml of 1% sucrose solution were provided. Then, after 23 h of water exclusion, 100 ml water and 100 ml of 1% sucrose solution (in opposite position compared to the second day) were provided. We calculated Sucrose Preference as:

$$ \frac{\mathrm{Sucrose}\ \mathrm{consumption}\left(\mathrm{g}\right)}{\left(\mathrm{Surcrose}\ \mathrm{consumption}+\mathrm{water}\ \mathrm{consumption}\right)\left(\mathrm{g}\right)}\times \left(100\%\right) $$

#### Open field test

After placation and adaptation to the environment, rats were put into open field separately. Infrared detectors were used to record indicators, including rearing time, total number into the center zone, the total number of line-crossing, the total number of rearing, the total number of rotations, and the total moving distance. Open field tests were applied before (baseline) and after (endpoint) CUMS procedure. Statistical analysis was performed on their difference. Before a new test start, we removed the feces and smell of the previous rats by 10% ethanol.

### Small RNA sequencing and data acquisition

Nucleus Accumbens from both sides were dissected from 6 rats (2 rats per groups).1.5 ml of NAc tissue was added into 800 μ Trizol reagent, and total RNA isolation was achieved based on the manufacturer instruction. mirVana™ miRNA Isolation Kit (Ca#t. AM1560 Austin TX, US) were used for extracting total RNA. miRNA-Sequencing was done on Illumina Hiseq-2000 using version 8 multiplexed single-read sequencing recipe in line with Illumina Solexa Small RNA Seq Protocol. Reverse transcription、amplification and cDNA purification were accomplished following Protocol. We used Qubit™ dsDNA HS and High Sensitivity DNA Chip for quality control of cDNA libraries. Raw reads were pre-processed by the fastx (fastx_toolkit-0.0.13.2) [59] software. We also removed adapters and low-quality reads. Effective reads were aligned to the miRbase database [21] 0 mismatches allowed) by CLC genomics_workbench 5.5 software. We also mapped our reads to ncRNA [60] database to remove other types of non-coding RNAs from the pool. Before downstream analyses, we summed up read counts of each miRNA across all samples and excluded lowest 25% of miRNAs, and miRNAs with 0 counts in any single sample, to remove the noise of low-expression miRNA.

### Differential expression analysis

Differential expression analyses were performed using DEGseq R package [61]. Since we didn’t have technical replicate, we used MA-plot-based method based on random sampling [61] to carry out differential expression analysis. As is recommended by the author of DEGseq [61], we used read counts instead of TPM for DEGseq analysis. We set the threshold of differential expression (DE) at |log2FC| > 1, and Benjamini–Hochberg FDR < 0.05. We compared miRNA expression in 1) control vs. depressed, 2) depressed vs. treatment, and 3) control vs. treatment comparison.

### Co-expression analysis of miRNA

Similar to classical weighted gene co-expression network analyses (WGCNA) [62], we performed a co-expression analysis of miRNA (namely WRCNA) using the WGCNA R package [34]. Expression matrix was transformed into log_10_(TPM) before analyses. We pick a soft threshold of 20 by *pickSoftThreshold* function. WRCNA identifies miRNAs that show co-expression patterns, which may reflect common biological functions, and groups them as “modules”. We calculate Module Eigengene as the first principle component of the module and is used as the weighted mean expression for miRNA within this module for downstream analysis. We performed module-trait correlation analyses regarding three traits: stress (rats from control group = 0, others =1), treatment (rats from treatment group = 1, others = 0) and anhedonia (rats from depression group = 1, others = 0). We calculated Pearson correlations between these traits and the weighted mean expression of each module (the module eigengene) and extracted miRNA in those modules that have a strong correlation with traits for downstream miRNA analyses.

### Target gene prediction

We predicted target genes of highlighted miRNAs by Targetscan7.2 [63]. We used these predicted target genes in network and pathway analyses. We filter putative target genes by cumulative weighted context++ scores [32] defined by Targetscan. Genes with lower context score have higher prediction reliability. For each interested miRNA lists, we pooled their predicted target genes together and applied downstream analyses on these integrated gene lists as a whole.

### Pathway analysis

We performed Gene Ontology (GO) [64], as well as KEGG [65] pathway, analyses on target genes of DE-miRNA lists (predicted targets of control Vs. depression DE-miR, depression vs. treatment DE-miR) and interested modules, revealed in WRCNA, on DAVID Bioinformatic tools [33]. We set a strict threshold of context score < − 0.5 to filter the putative target gene list. For depression versus treatment DE-miRNA lists, the threshold was set at context score < − 0.2, because it contained less miRNA (8) compared with other lists of interested miRNAs. Results were corrected for multiple tests by the Benjamini–Hochberg method. The threshold of significant enrichment was set as an adjusted *p* value< 0.05.

### Network analysis

We built the miRNA-gene regulation network using Cytoscape [66]. We filtered putative miRNA-gene regulation by different thresholds according to the number of interested miRNAs, so that the final networks could focus on hub molecules more explicitly. Since miRNAs from depression vs. treatment DE-miRNA list has relatively less predicted targets when we applied pathway analyses to them, we chose a relatively loose threshold (context score < − 0.4) to filter miRNA-gene interaction; for other lists of miRNAs, we set the threshold of context score at − 0.6. Gene nodes with only 1 miRNA partner were removed from the network.

### Statistical analysis

For results of behavior tests, we applied one-way ANOVA. We compared the difference of open field test data between endpoint and baseline in three groups, to get rid of the influence of difference at baseline. Since the experimental data were tested and basically accorded with the normal distribution, we chose the Least Significant Difference method for multi-comparison. The statistic was done by PASW Statistics 18.0. For bioinformatic analyses, statistical methods were described above, and were achieved in R3.4.2 (R Core Team (2017)) by corresponding packages.

## Supplementary information

**Additional file 1.** Behavioral tests results. a: Result for sucrose preference test triweekly. b: Weight of rats in three groups at different times.

**Additional file 2.** Overview of sequence results. Bagplots showing the composition of different kinds of small RNA, grouped by length of aligned reads.

## Data Availability

Clean count matrix, filtered TPM matrix and all R codes for analysis were stored in GitHub (https://github.com/WeiCSong/NAc_miR).

## Abbreviations

CUMS: Chronic unpredictable mild stress
DE: Differential expressing
GO-BP: Gene Ontology-Biological pathway
KEGG: Kyoto encyclopedia of genes and genomes
MDD: Major Depressive Disorder
miRNA: MicroRNA
NAc: Nucleus Accumbens
WRCNA: Weighted miRNA co-expression network analysis
