# Supplementary figures and images for: Expression alteration of microRNAs in Nucleus Accumbens is associated with chronic stress and antidepressant treatment in rats
Source: BMC Med Inform Decis Mak. 2019 Dec 19;19(Suppl 6):271. doi: 10.1186/s12911-019-0964-z (PMC6921443; doi:10.1186/s12911-019-0964-z)

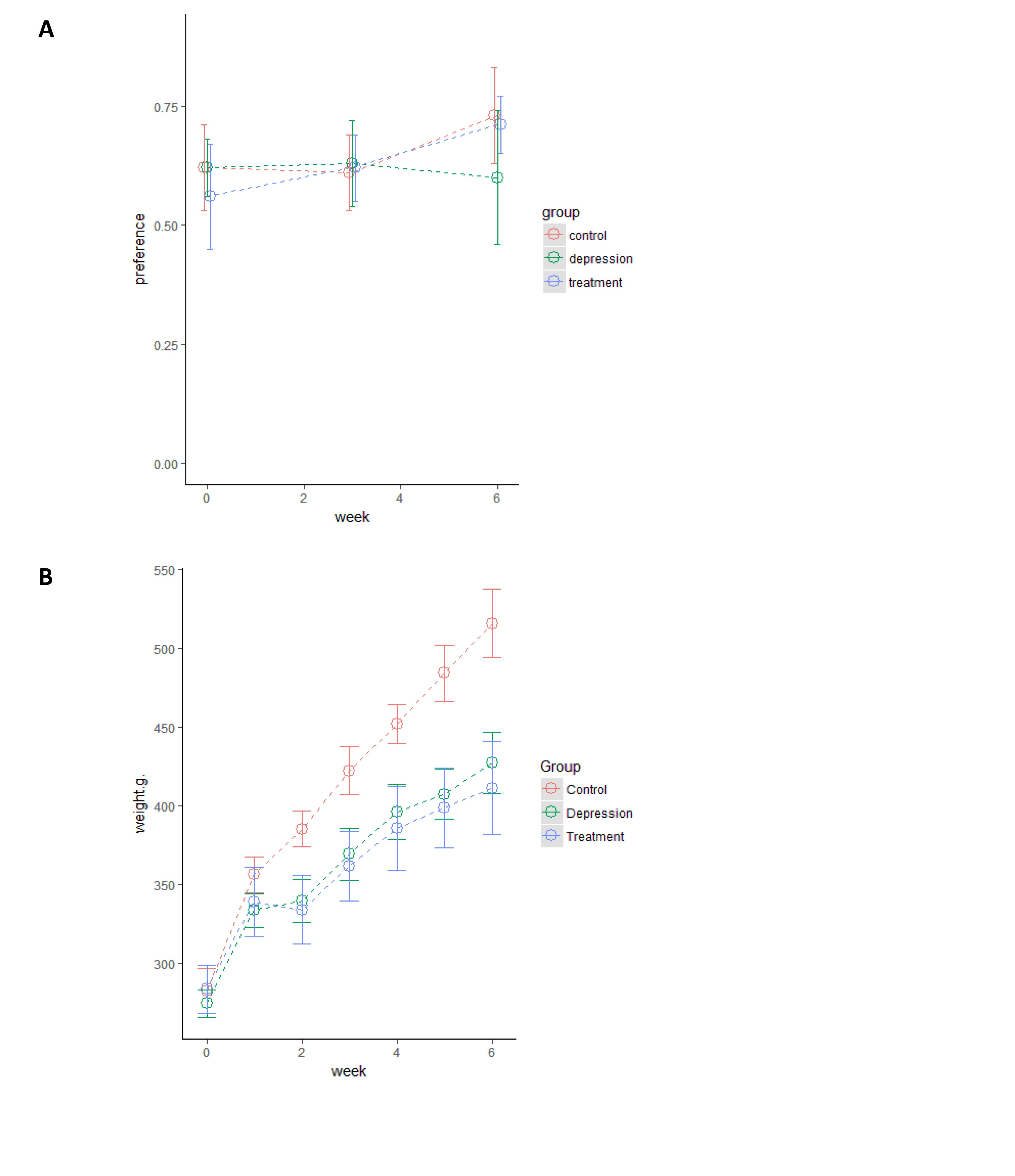

Supplement: Supplementary file 1 — Additional file 1. Behavioral tests results. a: Result for sucrose preference test triweekly. b: Weight of rats in three groups at different times. [file 12911_2019_964_MOESM1_ESM.jpg]

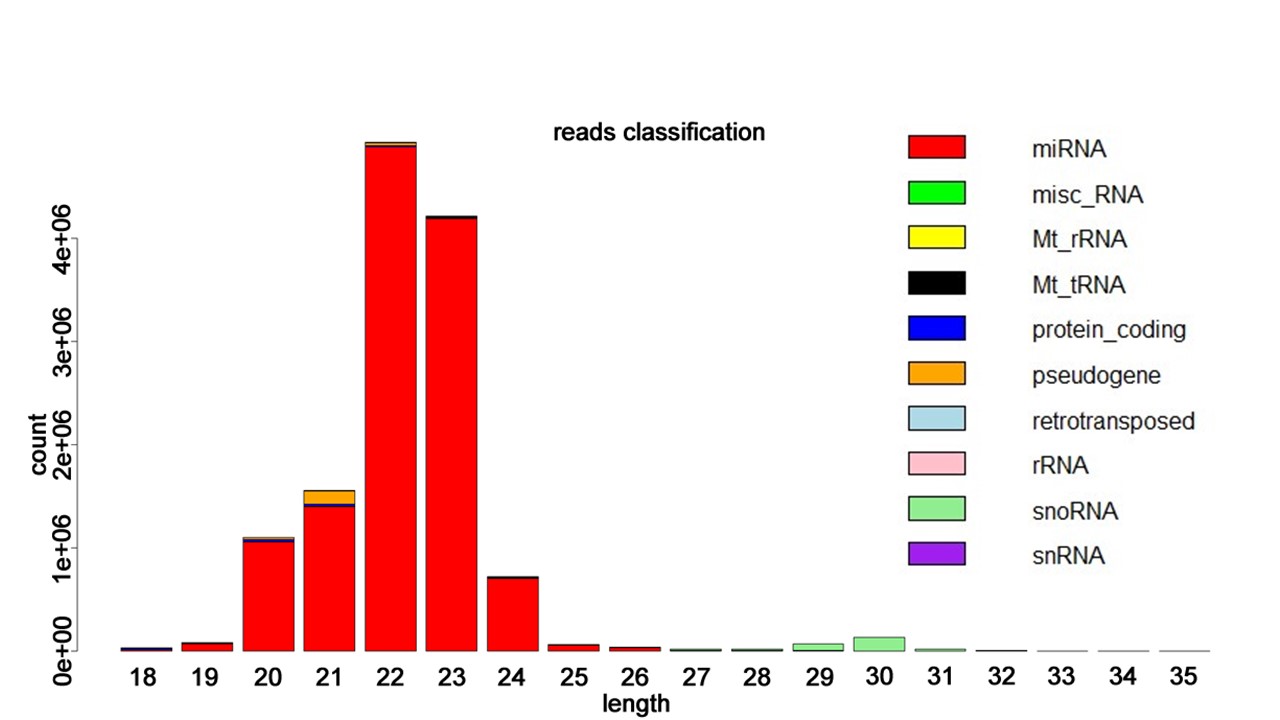

Supplement: Supplementary file 2 — Additional file 2. Overview of sequence results. Bagplots showing the composition of different kinds of small RNA, grouped by length of aligned reads. [file 12911_2019_964_MOESM2_ESM.jpg]
